# The efficacy and safety of IL-13 inhibitors in atopic dermatitis: A systematic review and meta-analysis

**DOI:** 10.3389/fimmu.2022.923362

**Published:** 2022-07-27

**Authors:** Yan Zhang, Danrong Jing, Jun Cheng, Xiang Chen, Minxue Shen, Hong Liu

**Affiliations:** ^1^ Department of Respiratory Medicine, Xiangya Hospital, Central South University, Changsha, China; ^2^ Department of Dermatology, Xiangya Hospital, Central South University, Changsha, China; ^3^ National Clinical Research Center for Geriatric Disorders, Xiangya Hospital, Central South University, Changsha, China; ^4^ Department of Spine Surgery, The Third Xiangya Hospital of Central South University, Changsha, China; ^5^ Department of Social Medicine and Health Management, Xiangya School of Public Health, Central South University, Changsha, China

**Keywords:** atopic dermatitis, interleukin-13 inhibitor, efficacy, safety, meta-analysis

## Abstract

**Background:**

Several clinical trials have evaluated the efficacy and safety of interleukin-13 (IL-13) with lebrikizumab and tralokinumab in patients with moderate to severe atopic dermatitis (AD). However, the safety and efficacy of IL-13 inhibitors as a potent biologic for AD remain elusive.

**Objective:**

To assess the efficacy and safety of IL-13 inhibitors in moderate to severe AD.

**Method:**

Randomized clinical trials (RCTs), comparing IL-13 inhibitors vs placebo treatment in patients with moderate to severe AD, were identified from public database from its inception to November 9^th^, 2021. The study was registered in PROSPERO (CRD42021254920).

**Results:**

Six studies reporting 7 RCTs involving 2946 patients with moderate-to-severe AD were included for the pooled analysis. Compared with placebo, antagonizing IL-13 with lebrikizumab and tralokinumab showed a greater improvement in percentage change of EASI (MD -20.37, 95%CI -32.28, -8.47), and a larger proportion of patients achieving numerical rating scale (NRS) with more than 4-points improvement (RR 1.59, 95%CI 1.23, 2.05). Additionally, IL-13 inhibitors also improved impaired dermatology life quality index (DLQI) (MD -14.49, 95%CI -19.23, -9.75). In terms of safety, both lebrikizumab and tralokinumab were well tolerated, with the except that they were linked to an increased risk of conjunctivitis compared to placebo treatment.

**Conclusion:**

Antagonizing IL-13 with lebrikizumab and tralokinumab have demonstrated encouraging clinical efficacy against moderate-to-severe AD with excellent safety profile, albeit they did come with a higher risk of conjunctivitis than placebo treatment.

**Systematic Review Registration:**

https://www.crd.york.ac.uk/prospero/, identifier ID=CRD42021254920.

## Introduction

Atopic dermatitis (AD) is a common chronic, relapsing, and inflammatory skin disease, characterized by recurrent eczematous skin lesions and intense pruritus. It affects approximately 20% of children and 3% of adults worldwide (1–3), resulting in accompanying phenomenon such as anxiety, depression, and sleep disturbance. Children with AD usually have a significant burden of daily activities and studies, and might face with an unfriendly environment at school (4). Adults with AD could also results in work absenteeism (1). These lead to a considerable financial and medical burden on society (5, 6). However, the current topical treatments are not always satisfactory and effective in achieving disease control in individuals with moderate to severe AD.

The treatment options for moderate to severe AD are limited to phototherapy, systemic immunomodulatory agents, or immunotherapy. Nevertheless, most of these treatments are recommended for short-term use and require rigorous follow-up, as long-term usage can result in a variety of adverse effects (7, 8). Therefore, effective and safe therapeutics for AD, especially for moderate-to-severe AD, are essential and critical. Recent years, biological therapy in AD, including targeted biological therapeutics (e.g., anti-IL-4Ra (dupilumab) (9), Janus kinase (JAK) inhibitors (10), and topical phosphodiesterase-4 (PDE-4) inhibitors (11)), have been shown to be promising possibilities for AD therapy. IL-13 has been known as an key inflammatory mediator in allergic responses, pruritus, skin barrier dysfunction, skin thickening and inflammation in AD (12, 13), and its gene polymorphisms are associated with an increased risk of AD (14). IL-13 expression in lesioned tissues, serum IL-13 levels and circulating T cells that produce IL-13 are significantly elevated in patients with AD compared to the healthy controls, and elevated IL-13 level is positively correlated with disease severity (15–17), indicating that IL-13 could be a potential therapeutic target for patients with AD.

Lebrikizumab and tralokinumab, as novel, high-affinity and monoclonal antibodies that selectively antagonizes IL-13, has been shown a promising effect in patients with moderate to severe AD (18, 19). Recently, tralokinumab was just approved by FDA for the treatment of moderate-to-severe atopic dermatitis in early 2022. However, the safety and efficacy of IL-13 inhibitors as a potent biologic for AD have not been comprehensively analyzed.

## Methods

### Search strategies

The study was registered in PROSPERO (ID=CRD42021254920) (https://www.crd.york.ac.uk/prospero/), and conducted based on the Preferred Reporting Items for Systematic Reviews and Meta-Analyses (PRISMA) guidelines (20). Changes from the protocol were mentioned in **Supplementary Methods**. Electronical databases including PubMed, Embase, Cochrane Central Register of Controlled Trials were searched from its inception to November 9^th^, 2021. Unpublished clinical trials on the website of ClinicTrials. Gov was searched as well. A combination of the term “atopic dermatitis” or “atopic eczema” and “anti-interleukin-13” or “anti-IL-13” or “lebrikizumab” or “LY3650150” or “tralokinumab” or “GSK679586” or “CAT-354” was used for the search strategies. The detailed search strategies used in Embase and PubMed are provided in **Supplementary Table 1**. The inclusion criteria were: (1) individuals with moderate to severe AD who did not receiving lebrikizumab or tralokinumab before; (2) RCT; (3) studies that compared interventions between IL-13 inhibitors and control/placebo; (4) studies that provided any interesting outcome, such as disease severity, quality of life and adverse events. The detailed search strategies used in the study are provided in **Supplementary Table 1**. Both Dr. Y.Z. and Dr. D.J. independently assessed the eligibility of each study, and any disagreement would be resolved by consensus with a third investigator (Dr. J. C.). Only RCT comparing IL-13 inhibitors and vehicle or placebo in patients with moderate to severe AD were included regardless of age, gender, race/ethnicity, dosages, and duration of administration. Non-RCT, observational or retrospective studies, nonhuman studies, duplicates, conference abstracts, and studies without outcomes of interest were excluded.

### Data extraction

Data regarding the first author, publication year, ClinicalTrials.gov identifier, number of participants, age, diagnosis criteria and intervention details and outcomes of interest were extracted with a standardized form.

The efficacy outcomes of interest in the study were assessed by the following parameters: (1) Percentage changes of Eczema Area and Severity Index (EASI) score from baseline; (2) proportion of patients achieving EASI score of greater than or equal to 75% from the baseline: [(baseline EASI – follow-up EASI)/baseline EASI × 100% ≥75%]; (3) proportion of patients achieving the Investigators’ Global Assessment (IGA) score of clear (0) or almost clear (1) with an improvement of more than 2 grades from the baseline; (4) proportion of patient with a reduction of pruritus numerical rating scale (NRS) more than 4 points; (5)and improvement of dermatology life quality index (DLQI).

The safety outcomes included at least one adverse effect (AE), at least one serious AE, treatment discontinuation owing to AEs, upper respiratory tract infection, headache, conjunctivitis, and injection-site reaction. When the desired outcomes were only presented in the figures, Image J software would be used to quantify the data from a graph (National Institutes of Health; https://imagej.nih.gov/ij/).

### Assessment of risk of bias

The risk of bias for the included RCTs were evaluated independently by two reviewers (Y.Z. and D.J.) based on the Cochrane Collaboration’s assessment tool. Any disagreement would be resolved by discussion and consensus with a third reviewer (J.C).

### Assessment of publication bias

The funnel plot and Egger test were used to assess possible publication bias.

### Statistical analysis

All meta-analysis in the study were synthesized with random-effects model using the Review Manager 5.4 and Stata 15.0, since this model enables the generalizability of results by assuming that the included studies are a random sample of effect sizes and by considering variability both of within- and between-study. Heterogeneity was evaluated according to the Cochran Q test and I² statistic. Mean difference (MD) and risk ratio (RR) with 95% confidence interval (CI) were used to present the effect sizes for continuous and dichotomous data respectively. If one study had more than one treatment group for IL-13 antibody, all intervention groups would be combined into one group based on the Cochrane handbook. A P value less than 0.05 was accepted statistically significant.

## Results

### Search results and characteristics of included studies

**Figure 1:**
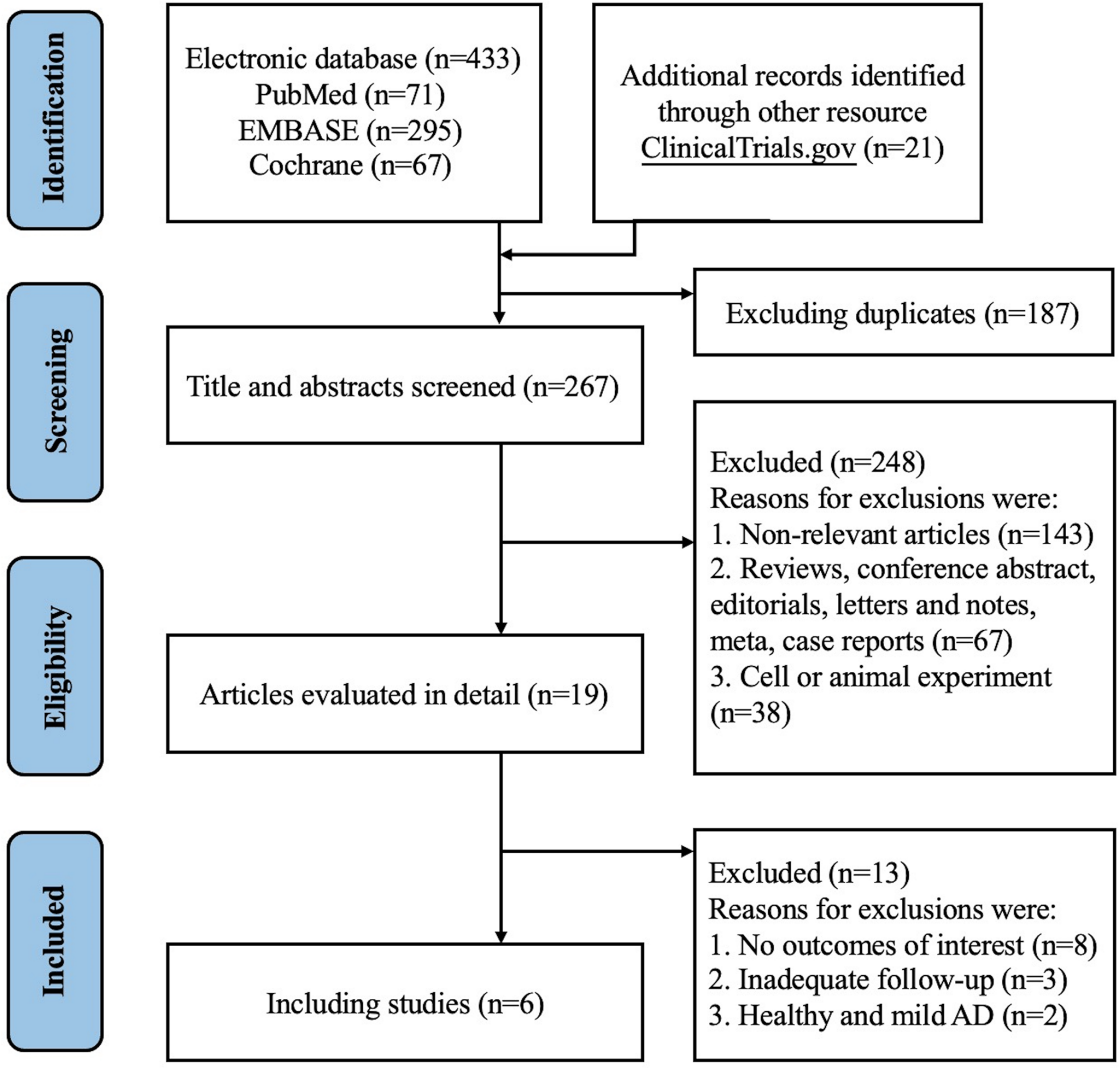
PRISM 2020 flow diagram for new systematic reviews which included searches of databases and registers only.

We initially identified 433 records from PubMed, Embase and CENTRAL databases (**eTable 1**), and 21 records from ClinicalTrials.gov as summarized in **Figure 1**. Of these, 187 records were duplicates and 248 records were excluded after titles and abstracts were screened, and 13 records were excluded after full text screening. Thus, a total of 6 studies (18, 19, 21–24) with 7 randomized, double-blind, multicenter, placebo-controlled RCTs involving 2946 cases with moderate-to-severe AD were finally included for meta-analysis, including 2 RCTs of Lebrikizumab and 5 RCTs of Tralokinumab. All the included studies used subcutaneous placebo as the comparator, and treatment duration varied from 12 weeks to 16 weeks. The detailed features of each included study are summarized and presented in **Table 1**. Adult patients with moderate to severe AD were recruited in all of included studies, which were published from 2018 to 2021. Risk of bias for all the RCTs were assessed and summarized in **eFigure 1** and **eTable 2** and showed high quality in all included RCTs. Only 1 study (21) did not report the detailed description of random sequence generation, allocation concealment, blinding of participants and health care personnel, and blinding of outcome assessment.

**Table 1:** Characteristics of the included studies (RCTs).

Study(year)	Clinical trial identified	Severity of AD	Participant characteristics						TreatmentDosage/frequency	Durationof treatment	Outcomes
			Treatment group			Placebo group					
			No. of participants/no. of males	Age (y)	Race (%)	No. of participants/no. of males	Age (y)	Race (%)			
E. Guttman-Yassky et al. (2020) (18)	NCT 03443024	Moderate to severe	228/86	38.7 ± 17.3	White (52.2) Black or African American (33.8) American Indian or Alaskan native (1.3) Asian (9.2) Multiple or other (3.5)	52/28	42.2 ± 18.2	White (50.0) Black or African American (30.8) American Indian or Alaskan native (0.0) Asian (11.5) Multiple or other (7.7)	Lebrikizumab (125mg Q4W, 250mg Q4W or 250 mg Q2W) or placebo	16 wk	EASI%, EASI-75, NRS improvement>4, DLQI
E. L. Simpson et al. (2018) (21)	NCT 02340234	Moderate to severe	156/100	35.3 ± 12.4	White (73.7) Asian (23.7) Other (25.6)	53/36	38.7 ± 13.2	White (66.0) Asian (30.2) Other (3.8)	Lebrikizumab (125mg SD, 250mg SD or 125 mg Q4W) or placebo	12 wk	EASI%, EASI-75, IGA 0/1
J. I. Silverberg et al. (2021) (19)	NCT 03363854	Moderate to severe	253/125	37.0 (28.0-52.-0)	White (80.2) Black or African American (9.1) Asian (6.7) Native Hawaiian or other Pacific Islanfer (0.4) Other (3.6)	127/84	34.0 (24.0-50.0)	White (66.9) Black or African American (9.4) Asian (18.9) Native Hawaiian or other Pacific Islanfer (0.8) Other (3.9)	Tralokinumab (300mg Q2W+TCS) or placebo	16 wk	EASI%, EASI-75, IGA 0/1, NRS improvement>4, DLQI
A. Wollenberg et al. ECZTRA1 (2021) (22)	NCT 03131648	Moderate to severe	603/351	37.0 (27.0-48.0)	White (70.6) Black (6.8) Asian (19.9) Other or missing data (2.7)	199/123	37.0 (26.0-49.0)	White (69.4) Black (9.0) Asian (20.1) Other or missing data (1.5)	Tralokinumab (300mg Q2W) or placebo	16 wk	EASI%, EASI-75, IGA 0/1, NRS improvement>4, DLQI
A. Wollenberg et al. ECZTRA2 (2021) (22)	NCT 03160885	Moderate to severe	593/359	34.0 (25.0-48.0)	White (63.1) Black (7.3) Asian (26.0) Other or missing data (3.7)	201/114	30.0 (23.0-46.0)	White (61.2) Black (8.5) Asian (25.9) Other or missing data (4.5)	Tralokinumab (300mg Q2W) or placebo	16 wk	EASI%, EASI-75, IGA 0/1, NRS improvement>4, DLQI
A. Wollenberg et al. (2019) (23)	NCT 02347176	Moderate to severe	153/88	37.3 ± 14.5	Asian (22.9) Black or African American (13.7) White (61.4) Other (0.7)	51/22	39.4 ± 14.5	Asian (19.6) Black or African American (15.7) White (60.8) Other (1.9)	Tralokinumab (45, 150 or 300mg Q2W) or placebo	12 wk	EASI%, IGA 0/1, DLQI
Gutermuth J. et al. (2021) (24)	NCT 03761537	Severe	140/82	33.0 (25.5-47.0)	White (97.9) Black or African American (0) Asian (0) Other (2.1)	137/83	34.0 (26.0-45.0)	White (98.5) Black or African American (0.7) Asian (0.7) Other (0)	Tralokinumab (300 mg Q2W+TCS or placebo)	16 wk	EASI%, EASI-75, NRS improvement>4, DLQI

### Efficacy outcomes

#### Clinician’s assessed disease severity

**Figure 2:**
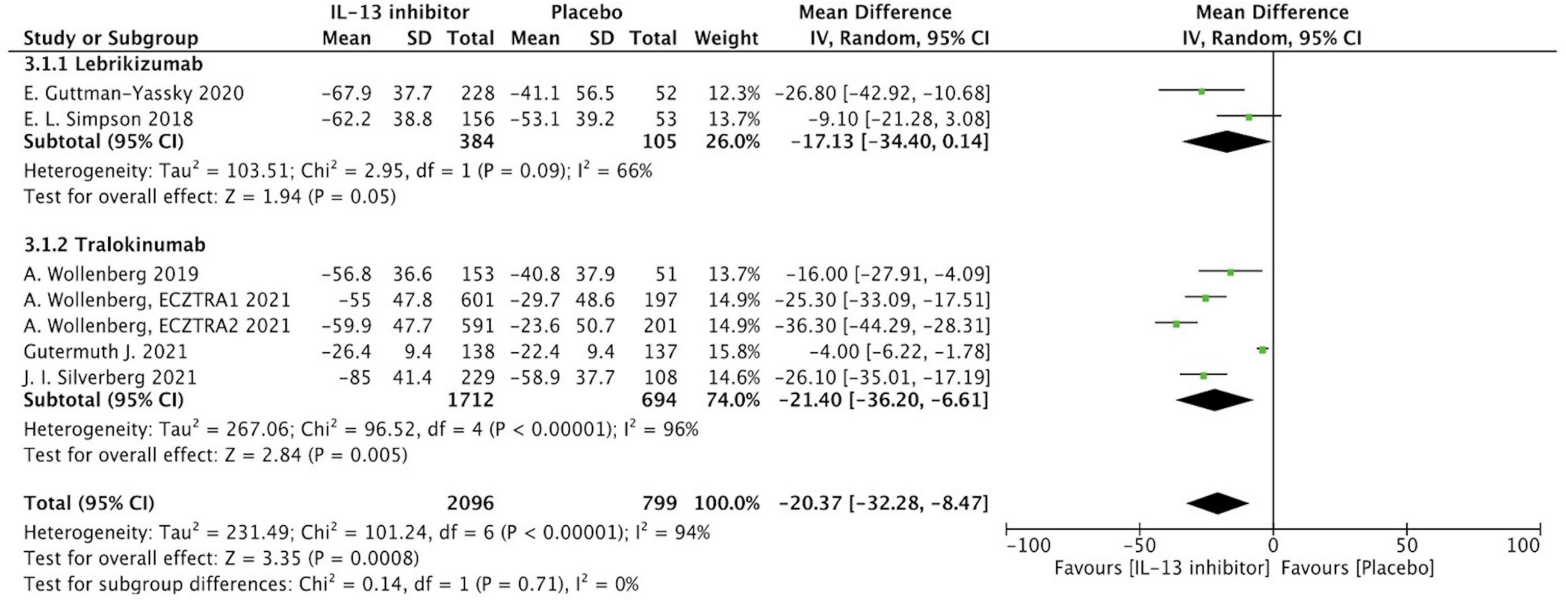
Forest plot for the percentage improvement of EASI score at the end of antagonizing IL-13 treatment.

Percentage of EASI score improvement at end of treatment (EOT) were extracted from 7 RCTs to determine the clinical efficacy of IL-13 treatment in AD patients. In AD patients, IL-13 treatment resulted in a substantial improvement in percentage change of EASI score when compared to placebo controls (MD -20.37, 95%CI -32.28, -8.47, P=0.0008) (**Figure 2**). However, these studies exhibited a significant heterogeneity, and subgroup analysis based on molecular subtypes of lebrikizumab and tralokinumab showed comparable beneficial effects and reduced heterogeneity. Additionally, EASI-75 at the EOT were extracted from 6 RCTs, and the meta-analysis indicated antagonizing IL-13 with lebrikizumab and tralokinumab had a larger proportion of moderate to severe AD patients achieving EASI-75 (**eFigure 2**). According to stratified analysis on different timepoints, antagonizing IL-13 with either lebrikizumab or tralokinumab had a greater proportion of patients achieving EASI-75 as early as week 4 [RR 2.09, 95%CI 1.24, 3.53), P=0.006] compared to the placebo controls, but mitigated with time-dependent effect from week 8 (35.6%) to week 16 (38.6%) [RR 1.83, 95%CI 1.35, 2.49), P=0.0001] (**eTable 3**).

IGA response at the EOT was analyzed in 6 RCTs, and the results demonstrated that antagonizing IL-13 resulted in a higher proportion of patients with IGA improvement of clear or almost clear (RR 1.76, 95%CI 1.44, 2.15, **eFigure 3**). However, the stratified analysis based on different timepoints showed a time-exposure effect of IGA response from week 4 (7.3%) to week 16 (24.1%) (**eTable 3**).

#### Patients reported outcomes

**Figure 3:**
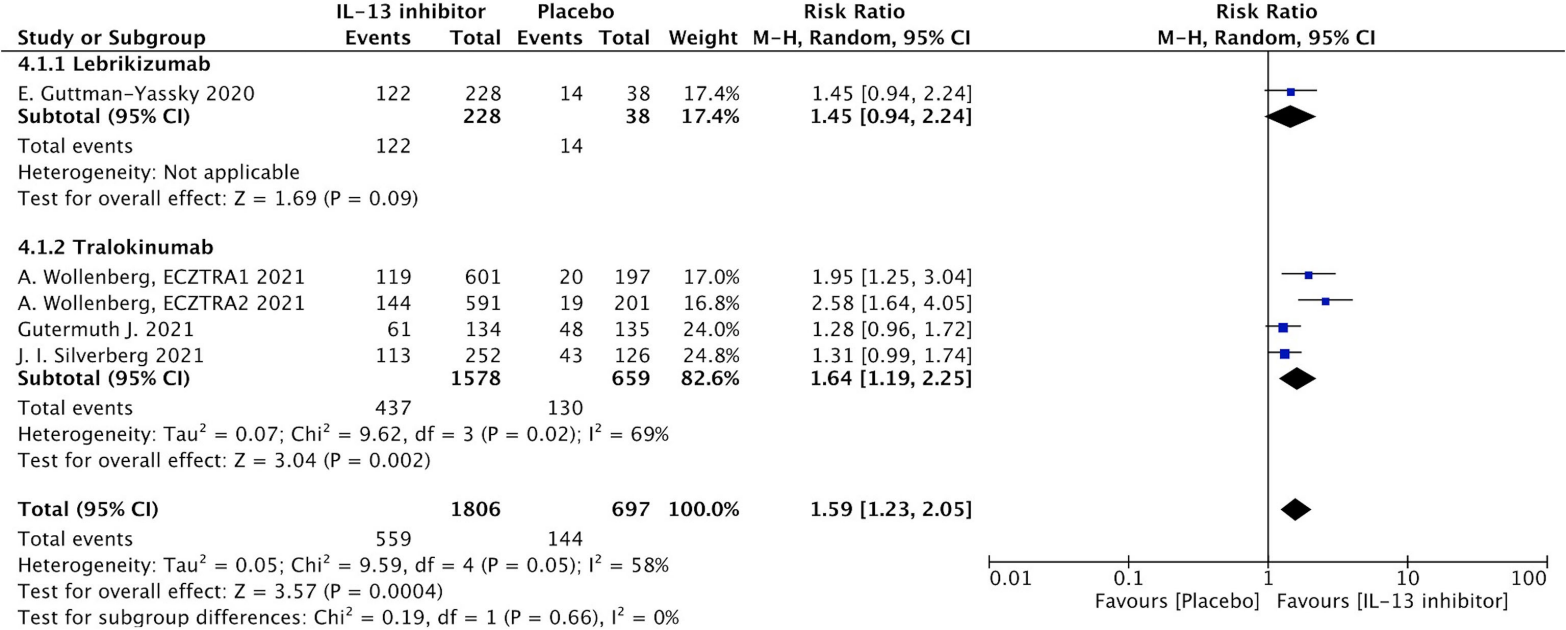
Forest plot for the proportion of patients achieving NRS score with more than 4-points daily improvement at the end of antagonizing IL-13 treatment.

**Figure 4:**
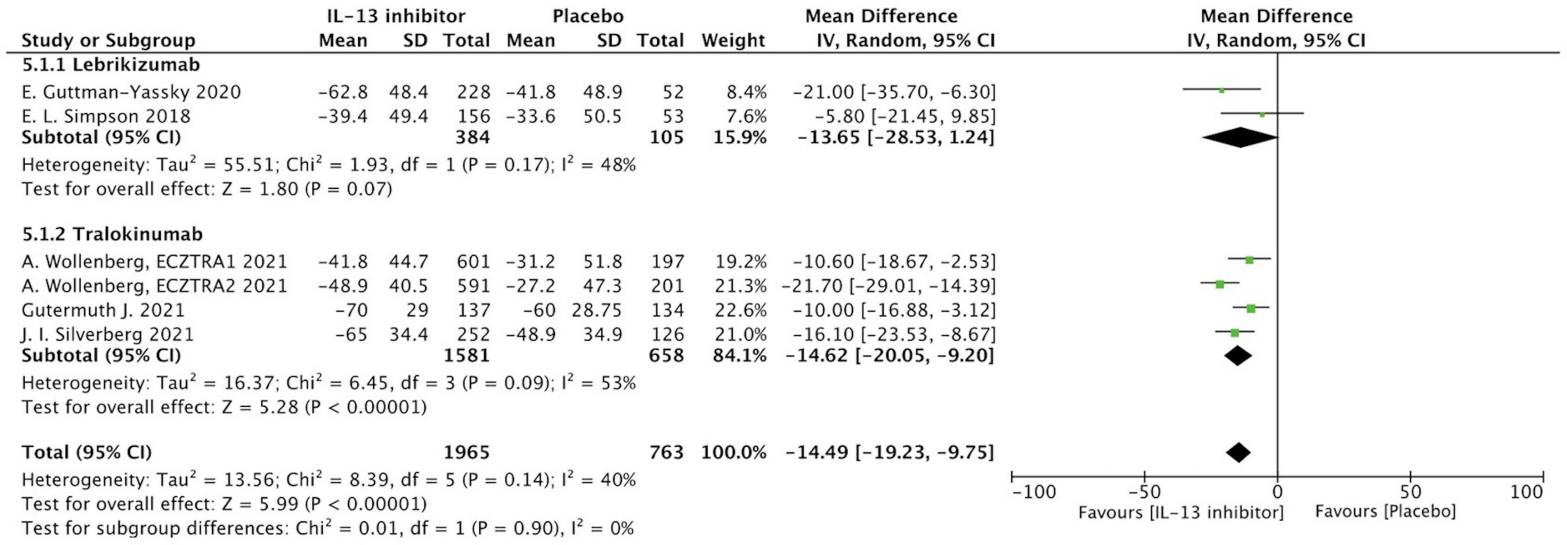
Forest plot for the improvement of DLQI at the end of antagonizing IL-13 treatment.

NRS is a relative subjective parameter to reflect itch from patients, and the proportion of patients achieving NRS score with more than 4-points daily improvement was higher in IL-13 inhibitor group compared to placebo group (RR 1.59, 95%CI 1.23, 2.05, **Figure 3**).The therapeutic effect was further supported by relatively subjective and patients-reported outcomes with significant improvement of DLQI (MD -14.49, 95%CI -19.23, -9,75, **Figure 4**), indicating IL-13 inhibitor could improve the impaired life quality caused by AD.

#### Safety

The treatment with lebrikizumab or tralokinumab had excellent safety profiles with no significant differences with respect to the risk of the following adverse events: at least one AE, at least one serious AE, discontinuation due to AEs, headaches, and injection-site reaction. However, antagonizing IL-13 with tralokinumab and lebrikizumab was accompanied with increased risk for conjunctivitis (RR 2.318, 95%CI 1.471, 3.652, P<0.001) compared to the placebo (**Table 2**). The subgroup analysis showed antagonizing IL-13 with tralokinumab, but not lebrikizumab, was accompanied by increased risk for conjunctivitis (RR 2.453, 95% 1.479, 4.068, P<0.001) compared to the placebo (**Table 2**). However, the prevalence of conjunctivitis as AE was comparable in either lebrikizumab (6.3%) and tralokinumab group (6.2%). The difference regarding the “significance” conjunctivitis between tralokinumab and lebrikizumab may be, to a large extent, due to the greater number of patients in the tralokinumab group.

**Table 2:** AEs associated with lebrikizumab and tralokinumab.

	Events (%)				Events (%)				Events (%)			
AEs	Combined group	Placebo	RR, random(95% CI)	*P*	Lebrikizumab	Placebo	RR, random(95% CI)	*P*	Tralokinumab	Placebo	RR, random(95% CI)	*P*
At least 1 AE	1336/1983 (67.4)	457/678 (67.4)	0.986 (0.935-1.041)	0.625	231/384 (60.2)	59/105 (56.2)	1.071 (0.748-1.532)	0.709	1105/1599 (69.1)	398/573 (69.5)	0.979 (0.926-1.035)	0.464
At least 1 serious AE	49/1983 (2.5)	22/678 (3.2)	0.715 (0.451-1.134)	0.153	9/384 (2.3)	4/105 (3.8)	0.615 (0.186-2.037)	0.427	40/1599 (2.5)	18/573 (3.1)	0.729 (0.441-1.205)	0.216
Treatment discontinuation due to AE	50/1983 (2.5)	18/678 (2.7)	0.938 (0.568-1.549)	0.803	10/384 (2.6)	1/105 (1.0)	2.734 (0.346-21.603)	0.340	40/1599 (2.4)	17/573 (3.0)	0.838 (0.495-1.420)	0.511
Upper respiratory tract infection	113/1827 (6.2)	30/625 (4.8)	1.188 (0.839-1.681)	0.330	17/228 (7.5)	3/52 (5.8)	1.292 (0.365-4.574)	0.691	96/1599 (6.0)	27/573 (4.7)	1.171 (0.814-1.685)	0.394
Headache	77/1827 (4.2)	26/625 (4.2)	0.971 (0.676-1.394)	0.872	8/228 (3.5)	3/52 (5.8)	0.608 (0.156-2.371)	0.474	69/1599 (4.3)	23/573 (4.0)	1.006 (0.690-1.466)	0.974
Conjunctivitis	113/1830 (6.2)	15/627 (2.4)	2.318 (1.471-3.652)	**<0.001**	24/384 (6.3)	4/105 (3.8)	1.641 (0.557-4.832)	0.369	89/1446 (6.2)	11/522 (2.1)	2.453 (1.479-4.068)	**<0.001**
Injection-site reaction	52/1983 (2.6)	16/678 (2.4)	1.111 (0.639-1.933)	0.709	15/384 (3.9)	2/105 (1.9)	2.051 (0.462-9.110)	0.345	37/1599 (2.3)	14/573 (2.4)	0.947 (0.508-1.765)	0.864

#### Publication bias

The funnel plot was not performed to assess possible publication bias for less than 10 trials, but Egger tests showed a possible publication bias of percentage changes of EASI and NRS (P=0.038, 0.04, respectively), and other tests on EASI-75, IGA and DLQI did not show significant publications bias (P=0.090, 0.506, 0.887, respectively). These findings suggest that there is a publication bias in the limited number of studies.

## Discussion

A comprehensive systematic review and meta-analysis was conducted in the study to assess the efficacy and safety of IL-13 inhibitors in patients with moderate to severe AD. The findings indicated that IL-13 inhibitors, either lebrikizumab or tralokinumab, were well tolerated and associated with significant and rapid improvement in clinical severity of patients with moderate to severe AD, indicating that IL-13 inhibitors were a safe and effective therapy for patients with moderate to severe AD.

The efficacy of IL-13 inhibitors was assessed using numerous criteria in all of the pooled studies. EASI score and IGA for AD are two of them that are relatively objective, acknowledged, and canonical scales that have been examined by experienced investigators (25). The following efficacy outcomes were combined in this study: EASI improvement, proportion of patients achieving EASI-75 and IGA response were pooled analyzed, and the results indicated that IL-13 inhibitors were effective in reducing disease severity of AD. This therapeutic effect was further supported by relatively subjective and patients-reported outcomes with significant improvement of DLQI and NRS score with more than 4-points daily improvement. Stratified analysis based on the timepoints of EASI-75 and IGA response showed antagonizing IL-13 could control the disease severity quickly, and the overall response of EASI-75 ranged from 16.0% at week 4 and to 38.6% at week 16, and the overall response of IGA clear or almost clear ranged from 7.3% at week 4 to 24.1% at week 16, indicating that only a proportion of moderate-to-severe AD patients could effectively respond to IL-13 inhibitors. Despite antagonizing IL-13 with lebrikizumab and tralokinumab shows a prospective application potential, there remain a large proportion of patients who do not well respond to anti-IL-13 biologics, probably due to heterogeneity of disease. This therapeutic effect could be concluded that it, to some extent, was not superior to dupilumab treatment for moderate-to-severe AD in clinical trials and real-world settings (26), in which the pooled proportion of patients achieving EASI-75 was more than 50% after 16 weeks therapy. It also seems not to be superior to abrocitinib, a selective JAK1 and JAK2 inhibitor, for the treatment of moderate-to-severe AD, which reported a pooled proportion of patients achieving EASI-75 proximately up to 50% within 16 weeks therapy (27). However, the therapeutic effect of anti-IL-13 might be partially veiled by pooling different doses of lebrikizumab and tralokinumab together, which may result in heterogeneity and biased effect estimates as these medications can have substantial dose-response effects. In addition, Wollenber A and his colleagues tried to explore biomarker based on subgroup analysis, and found tralokinumab treatment exhibited greater improvements of EASI score and IGA response versus placebo in DPP-4-high and periostin-high subgroups than in the ITT population (22). A comparable impact was shown in tralokinumab treated asthmatic patients for reducing annual asthma exacerbation rate (AAER) (13). Thus, identifying potential biomarkers to distinguish the patients who are more likely to benefit from IL-13 mediated biologics is critical.

Antagonizing IL-13 with both lebrikizumab and tralokinumab work quickly in AD, however, they have a certain threshold and ability of controlling and maintaining the remission of the disease, and they can’t further reduce the disease severity once that certain threshold is reached. In Wollenberg A’s study (22), the primary outcomes of diseases severity were assessed at week 16 in the initial treatment, after which some participants were re-randomized to treatment with continued tralokinumab Q2W, reduced frequency of tralokinumab Q4W or switched to placebo Q2W, and the maintenance outcomes were determined at week 52 during the maintenance treatment. The proportion of EASI-75 and IGA clear or almost clear gradually decreased from the continued tralokinumab treatment group to reduced tralokinumab or placebo groups, implying that the clinical response of antagonizing IL-13 treatment was difficult to sustain when IL-13 inhibitors were withdrawn. Moreover, the most recent ECZTEND study, which is a long-term extension study of tralokinumab, shows a persistent, progressive increase in the efficacy of tralokinumab after 16 weeks. And the safety profile of tralokinumab has also been proved with low AEs. In conjunction with our findings, it can be deliberately concluded that administration of IL-13 inhibitors with lebrikizumab and tralokinumab works quickly to control disease severity of AD, providing a promising alternative to systemic treatment for patients with moderate to severe AD. And tralokinumab could also provide maintenance effects and stable safety in a long term. However, further analysis of long-term efficacy and safety to lebrikizumab is still warranted.

Both lebrikizumab and tralokinumab were well tolerated across a wide range of medical conditions (13, 28). In the treatment of lebrikizumab and tralokinumab, there were no differences in any AE, any serious AE, and treatment discontinuation due to AE. Even so, patients who got tralokinumab had a significantly higher rate of conjunctivitis than those who received placebo; similar tendency of increased conjunctivitis did not reach a significance in lebrikizumab subgroup. However, the prevalence of conjunctivitis as AE was comparable in either lebrikizumab (6.3%) and tralokinumab group (6.2%). The difference regarding the “significance” conjunctivitis in the “safety” section between tralokinumab and lebrikizumab is to a large extent, due to the higher number of patients in the tralokinumab group (phase 3 included) vs. the lebrikizumab group (phase 3 not included). Long-term AEs, as well as real-word AEs, must be examined.

Currently, several biologics including dupilumab, monoclonal antibody that antagonizes both IL-4 and IL-13 signaling pathway, is approved and recommended as an alternative to systemic immunosuppressive treatment in patients with moderate-to-severe AD inadequately controlled by TCS. However, compared to IL-4, IL-13 expression is much higher and more frequently detected in the skin lesions of AD (15, 29), suggesting that IL-13 might also be a potential biological target besides IL-4 in AD. Furthermore, conjunctivitis, one of the most prevalent ocular comorbidities in AD (30), has also been reported as a considerable side effect in treatment with dupilumab (31), and biopsies from AD patients treated with dupilumab showed a substantial shortages of intraepithelial goblet cells (32). With the regard to safety, dupilumab-treated patients had a greater proportion of individuals with conjunctivitis as AE (up to 22% in clinical trials), which appeared less frequently in patients treated with either lebrikizumab (6.3%) or tralokinumab (6.2%), indicating a potential advantage over dupilumab (33). Therefore, there remains a tremendous unmet need for additional targeted systemic or biologic therapies with durable efficacy and safety profiles.

## Limitations

Regardless of the strengths of this systematic review and meta-analysis, there are some limitations. Firstly, high heterogeneity was detected in the pooled EASI and IGA response, therefore, the random-effects model was used for these meta-analyses to consider the heterogeneity caused by variability both of within-and between-studies. Secondly, multiple doses of lebrikizumab and tralokinumab were pooled together, which could lead to heterogeneity and biased effect estimates for substantial dose-response effects. Thirdly, the dose- and frequency-dependent response of lebrikizumab and tralokinumab, and role for reducing TCS treatment could not be analyzed due to the limited studies. Finally, majority of trials included in the study had short-term treatment durations, indicating long-term follow-up trials are needed.

## Conclusions

In summary, antagonizing IL-13 with either lebrikizumab or tralokinumab could significantly reduce the disease severity and improve the quality of life, thus providing a promising alternative option for AD. More powered studies would be warranted to evaluate the long-term and durable clinical response of IL-13 inhibitors, and to identify certain patients who were more likely to respond to IL-13 inhibitors, thus enabling more efficient management of Th2-mediated AD compared with currently available biologics.

## Data availability statement

The original contributions presented in the study are included in the article/**Supplementary Material**. Further inquiries can be directed to the corresponding author.

## Author contributions

YZ had the conception, collected and analyze the data, and wrote the manuscript. DJ collected and analyze the data, and wrote the manuscript. JC collected and analyze the data. XC had the conception and revised the manuscript. Ms had the conceptio , help the methods and revised the manuscript. HL had the conception and revised the manuscript. All authors contributed to the article and approved the submitted version.

## Funding

This work was supported by Natural Science Foundation of China (No.82100037 to YZ, No.82022060 to HL), National Key Research and Development Program of China (No.2019YFA0111600 to HL, No.2019YFE0120800 to HL), National Science Foundation for Post-doctoral Scientists of China (No. 2021TQ0375 to YZ, No. 2022M713538 to YZ), Hunan Outstanding Postdoctoral Innovative Talents Program (No.2021RC2018 to YZ), the Natural Science Foundation of Hunan Province for outstanding Young Scholars (No.2019JJ30040 to HL), and Youth Foundation of Xiangya Hospital (No.2020Q06 to YZ).

## Conflict of interest

The authors declare that the research was conducted in the absence of any commercial or financial relationships that could be construed as a potential conflict of interest.

## Publisher’s Note

All claims expressed in this article are solely those of the authors and do not necessarily represent those of their affiliated organizations, or those of the publisher, the editors and the reviewers. Any product that may be evaluated in this article, or claim that may be made by its manufacturer, is not guaranteed or endorsed by the publisher.

## Supplementary Material

Supplementary Figure 1 Assessment of risk bias of RCTs.

Supplementary Figure 2 Forest plot for proportion of patients achieving EASI-75 at the end of antagonizing IL-13 treatment.

Supplementary Figure 3 Forest plot for proportion of patients achieving PGA response at the end of antagonizing IL-13 treatment.
