# Supplementary figures and images for: The efficacy and safety of IL-13 inhibitors in atopic dermatitis: A systematic review and meta-analysis
Source: Front Immunol. 2022 Jul 27;13:923362. doi: 10.3389/fimmu.2022.923362 (PMC9364267; doi:10.3389/fimmu.2022.923362)

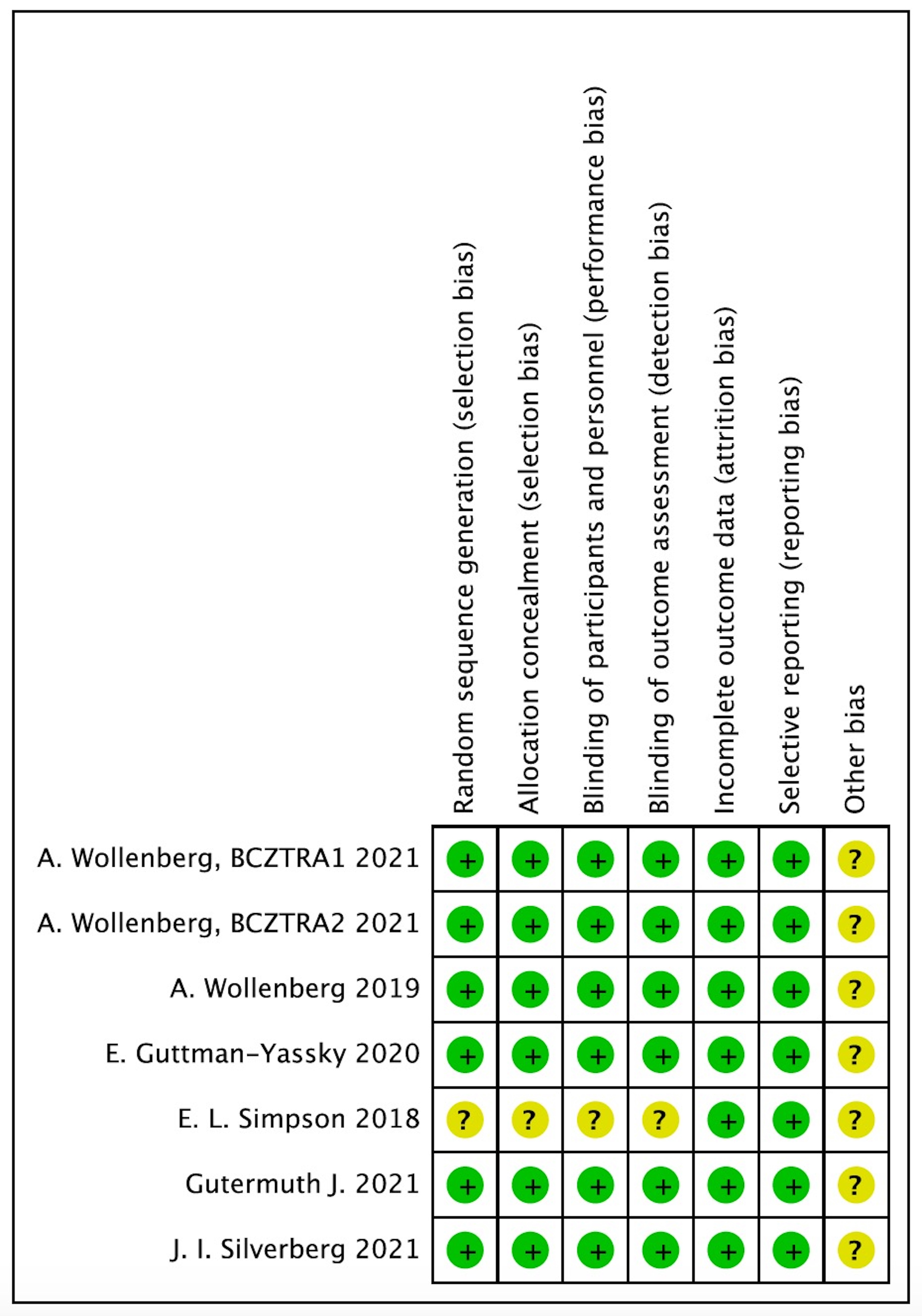

Supplement: Supplementary Figure 1 — Assessment of risk bias of RCTs. [file Image_1.jpeg]

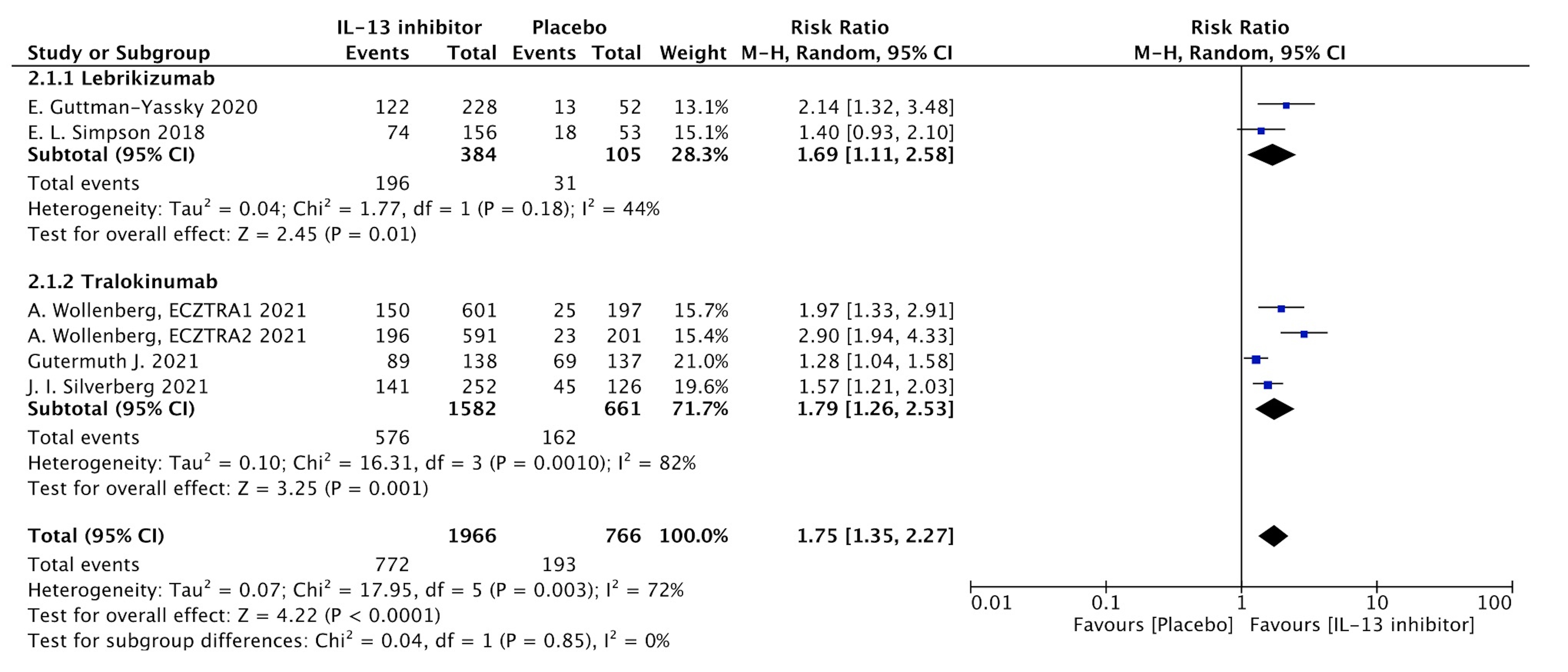

Supplement: Supplementary Figure 2 — Forest plot for proportion of patients achieving EASI-75 at the end of antagonizing IL-13 treatment. [file Image_2.jpeg]

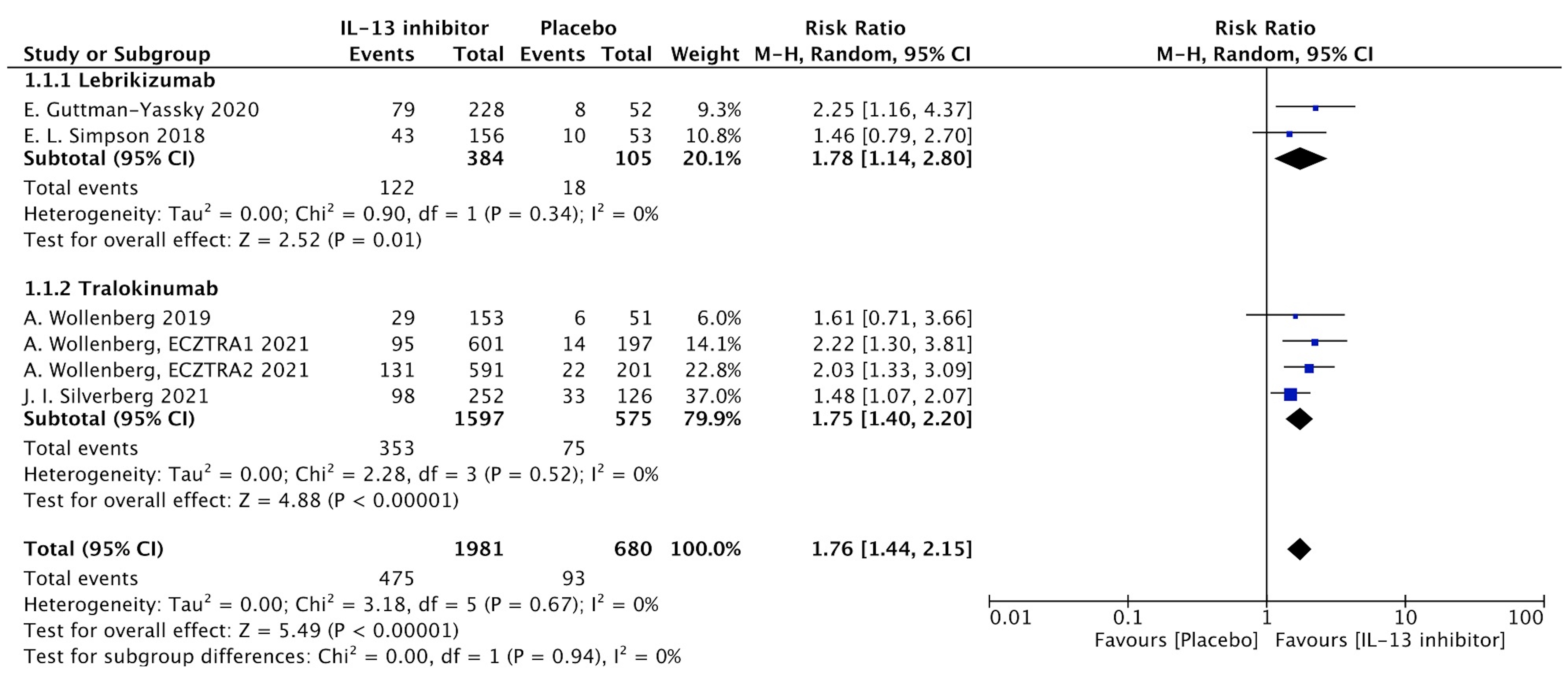

Supplement: Supplementary Figure 3 — Forest plot for proportion of patients achieving PGA response at the end of antagonizing IL-13 treatment. [file Image_3.jpeg]
