# Supplementary material for: The efficacy and safety of IL-13 inhibitors in atopic dermatitis: A systematic review and meta-analysis
Source: Front Immunol. 2022 Jul 27;13:923362. doi: 10.3389/fimmu.2022.923362 (PMC9364267; doi:10.3389/fimmu.2022.923362)
Supplement: Supplementary file 4 [file Table_1.docx]

**eTable 1. Search strategies of the study.**

| Search strategies in PubMed | | |
| --- | --- | --- |
| #1 | Atopic dermatitis | 31026 |
| #2 | Atopic eczema | 32561 |
| #3 | Anti-IL-13 | 216 |
| #4 | Anti-interlukin-13 | 0 |
| #5 | Lebrikizumab | 111 |
| #6 | LY3650150 | 0 |
| #7 | Tralokinumab | 107 |
| #8 | GSK679586 | 3 |
| #9 | CAT-354 | 114 |
| #10 | #1 OR #2 | 32156 |
| #11 | #3 OR #4 OR #5 OR #6 OR #7 OR #8 OR #9 | 355 |
| #12 | #10 AND #11 | 71 |
| Search strategies in Embase | | |
| #1 | Atopic dermatitis | 56918 |
| #2 | Atopic eczema | 52558 |
| #3 | Anti-IL-13 | 318 |
| #4 | Anti-interlukin-13 | 0 |
| #5 | Lebrikizumab | 668 |
| #6 | LY3650150 | 0 |
| #7 | Tralokinumab | 573 |
| #8 | GSK679586 | 5 |
| #9 | CAT-354 | 611 |
| #10 | #1 OR #2 | 57458 |
| #11 | #3 OR #4 OR #5 OR #6 OR #7 OR #8 OR #9 | 1163 |
| #12 | #10 AND #11 | 295 |
| Search strategies in Cochrane | | |
| #1 | Atopic dermatitis | 4982 |
| #2 | Atopic eczema | 2274 |
| #3 | Anti-IL-13 | 54 |
| #4 | Anti-interlukin-13 | 0 |
| #5 | Lebrikizumab | 86 |
| #6 | LY3650150 | 4 |
| #7 | Tralokinumab | 99 |
| #8 | GSK679586 | 5 |
| #9 | CAT-354 | 32 |
| #10 | #1 OR #2 | 4978 |
| #11 | #3 OR #4 OR #5 OR #6 OR #7 OR #8 OR #9 | 219 |
| #12 | #10 AND #11 | 67 |
| Search strategies in unpublished clinical trials | | |
| #1 | (Atopic dermatitis OR Atopic eczema) AND | 1084 |
| #2 | Atopic eczema | 960 |
| #3 | Anti-IL-13 | 2 |
| #4 | Anti-interlukin-13 | 0 |
| #5 | Lebrikizumab | 26 |
| #6 | LY3650150 | 9 |
| #7 | Tralokinumab | 28 |
| #8 | GSK679586 | 2 |
| #9 | CAT-354 | 10 |
| #10 | #1 OR #2 | 1084 |
| #11 | #3 OR #4 OR #5 OR #6 OR #7 OR #8 OR #9 | 59 |
| #12 | #10 AND #11 | 21 |
