# Supplementary material for: The efficacy and safety of IL-13 inhibitors in atopic dermatitis: A systematic review and meta-analysis
Source: Front Immunol. 2022 Jul 27;13:923362. doi: 10.3389/fimmu.2022.923362 (PMC9364267; doi:10.3389/fimmu.2022.923362)
Supplement: Supplementary file 5 [file Table_2.docx]

**eTable 2. The detailed assessment of bias risk**

| E. Guttman-Yassky et al^[17]^ | | |
| --- | --- | --- |
| Bias | Authors' judgement | Support for judgement |
| Random sequence generation (selection bias) | Low | Patients sequentially received a screening number assigned via iMedidata Rave (Medidata SolutionsWorldwide, Inc). |
| Allocation concealment (selection bias) | Low | Randomized, the patient’s screening number will be entered in the IWRS, and the patient will be assigned a patient study-drug kit. One kit will provide all the study drug for that patient based on the assigned treatment group. Patients will be randomized 3:3:3:2 to one of the following treatment groups. |
| Blinding of participants and personnel (performance bias) | Low | The sponsor (Dermira, Inc), investigators, study site personnel, and patients were blinded to treatment assignments, and blind integrity was maintained throughout the study. Blinded, coded kits with study drug in prefilled syringes and boxes masked the treatment assignments. |
| Blinding of outcome assessment (detection bias) | Low | Double-blinded, no description. |
| Incomplete outcome data (attrition bias) | Low | All relevant outcomes were described |
| Selective reporting (reporting bias) | Low | All relevant outcomes were described. |
| Other bias | Unclear | Unclear |

| E. L. Simpson et al^[21]^ | | |
| --- | --- | --- |
| Bias | Authors' judgement | Support for judgement |
| Random sequence generation (selection bias) | Unclear | Randomized, no description |
| Allocation concealment (selection bias) | Unclear | No description |
| Blinding of participants and personnel (performance bias) | Unclear | Blind, no description |
| Blinding of outcome assessment (detection bias) | Unclear | Blind, no description |
| Incomplete outcome data (attrition bias) | Low | All relevant outcomes were described |
| Selective reporting (reporting bias) | Low | All relevant outcomes were described |
| Other bias | Unclear | Unclear |

| J. I. Silverberg et al^[18]^ | | |
| --- | --- | --- |
| Bias | Authors' judgement | Support for judgement |
| Random sequence generation (selection bias) | Low | Randomization was performed using a using a computer-generated randomization schedule |
| Allocation concealment (selection bias) | Low | Treatment allocation was blinded to patients and  investigators (see Supporting Information). |
| Blinding of participants and personnel (performance bias) | Low | Double-blind, no description |
| Blinding of outcome assessment (detection bias) | Low | Double-blind, no description |
| Incomplete outcome data (attrition bias) | Low | All relevant outcomes were described. |
| Selective reporting (reporting bias) | Low | All relevant outcomes were described. |
| Other bias | Unclear | Unclear |

| A. Wollenberg et al^[22]^-ECZTRA1&ECZTRA2 | | |
| --- | --- | --- |
| Bias | Authors' judgement | Support for judgement |
| Random sequence generation (selection bias) | Low | Randomization was performed using a computer-generated randomization  schedule stratified by region and baseline disease severity |
| Allocation concealment (selection bias) | Low | The packaging and labelling of the IMPs contained no evidence of their identity. Since tralokinumab and placebo were visually distinct and not matched for viscosity, the IMP was handled and administered by a qualified, unblinded HCP at the site who was not involved in the management of trial subjects and who did not perform any of the assessments. |
| Blinding of participants and personnel (performance bias) | Low | Double blinded: Neither the subject nor any of the investigators or staff involved in the treatment or clinical evaluation and monitoring of the subjects were aware of the treatment received. |
| Blinding of outcome assessment (detection bias) | Low | Double-blind, no description |
| Incomplete outcome data (attrition bias) | Low | All relevant outcomes were described |
| Selective reporting (reporting bias) | Low | All relevant outcomes were described. |
| Other bias | Unclear | Unclear |

| A. Wollenberg et al^[23]^ | | |
| --- | --- | --- |
| Bias | Authors' judgement | Support for judgement |
| Random sequence generation (selection bias) | Low | An Interactive Web and Voice Response System (IWRS/IVRS) was used to randomize patients to a treatment group. |
| Allocation concealment (selection bias) | Low | Tralokinumab and placebo are visually distinct when stored in vials and are not matched for viscosity; however, they cannot be visually distinguished when drawn into a syringe. Therefore, to ensure appropriate blinding in the study, investigational product was handled by an unblinded investigational product manager at the site and was administered by an unblinded study team member who was not involved in the management of patients. |
| Blinding of participants and personnel (performance bias) | Low | This was a double-blind study in which patients, investigators, and sponsor staff were blinded to the treatment received. |
| Blinding of outcome assessment (detection bias) | Low | This was a double-blind study in which patients, investigators, and sponsor staff were blinded to the treatment received. |
| Incomplete outcome data (attrition bias) | Low | All relevant outcomes were described. |
| Selective reporting (reporting bias) | Low | All relevant outcomes were described. |
| Other bias | Unclear | Unclear |

| Gutermuth J. et al^[24]^ | | |
| --- | --- | --- |
| Bias | Authors' judgement | Support for judgement |
| Random sequence generation (selection bias) | Low | Randomization was performed using the interactive response technology stratified by prior cyclosporine A (CSA) use (yes/no), country (Germany: yes/no), and baseline disease severity (Investigator’s Global Assessment [IGA] score: 3/4). |
| Allocation concealment (selection bias) | Low | The blinded study drug was provided in coded kits in a non-sequential numbering system to ensure unblinding did not occur. As tralokinumab and the placebo are visually distinct, they were handled and administered by a qualified, unblinded healthcare professional at the site, who was not involved in the management or assessment of trial patients. |
| Blinding of participants and personnel (performance bias) | Low | Treatment allocation was blinded to patients and investigators. |
| Blinding of outcome assessment (detection bias) | Low | Double-blind, no description |
| Incomplete outcome data (attrition bias) | Low | All relevant outcomes were described. |
| Selective reporting (reporting bias) | Low | All relevant outcomes were described. |
| Other bias | Unclear | Unclear |
