# Supplementary material for: The efficacy and safety of IL-13 inhibitors in atopic dermatitis: A systematic review and meta-analysis
Source: Front Immunol. 2022 Jul 27;13:923362. doi: 10.3389/fimmu.2022.923362 (PMC9364267; doi:10.3389/fimmu.2022.923362)
Supplement: Supplementary file 6 [file Table_3.docx]

**eTable 3. Subgroup analyses of the efficacy of IL-13 inhibitor treatment in moderate to severe AD based on different time points.**

| Timepoints | IL-13 inhibitors | |  | Placebo |  | RR, 95%CI | | P value | | I² |
| --- | --- | --- | --- | --- | --- | --- | --- | --- | --- | --- |
|  | Events/Total participants (%) | |  | Events/Total participants (%) | |  |  |  |  |  |
| **EASI-75** |  |  |  |  |  |  |  |  |  |  |
| At week 4 | 292/1828(16.0%) | |  | 48/629(7.6%) |  | 2.09(1.24, 3.53) | | P=0.006 | | 64% |
| At week 8 | 502/1828(35.6%) | |  | 84/629(13.3%) |  | 1.94(1.35, 2.80) | | P=0.0003 | | 65% |
| At week 12 | 619/1828(33.9%) | |  | 110/629(17.5%) |  | 1.85(1.28, 2.68) | | P=0.001 | | 76% |
| At week 16 | 698/1810(38.6%) | |  | 175/713(24.5%) |  | 1.83(1.35, 2.49) | | P=0.0001 | | 77% |
| **IGA response** |  |  |  |  |  |  |  |  |  |  |
| At week 4 | 133/1828(7.3%) |  |  | 27/629(4.3%) |  | 1.75(0.99, 3.10) | | P=0.05 | | 35% |
| At week 8 | 281/1828(15.4%) | |  | 45/629(7.2%) |  | 2.28(1.35, 3.85) | | P=0.002 | | 60% |
| At week 12 | 414/1981(20.9%) | |  | 71/680(10.4%) |  | 2.15(1.29, 3.56) | | P=0.003 | | 73% |
| At week 16 | 403/1672(24.1%) | |  | 77/576(13.4%) |  | 1.81(1.45, 2.26) | | P<0.00001 | | 0% |
